# Molecular characterization of a novel Muscovy duck parvovirus isolate: evidence of recombination between classical MDPV and goose parvovirus strains

**DOI:** 10.1186/s12917-017-1238-6

**Published:** 2017-11-09

**Authors:** Jianye Wang, Jueyi Ling, Zhixian Wang, Yu Huang, Jianzhong Zhu, Guoqiang Zhu

**Affiliations:** 1 College of Veterinary Medicine, Yangzhou University, 48 Wenhui East Road, 225009 Yangzhou, Jiangsu Province People’s Republic of China; 2 Jiangsu Co-Innovation Center for Important Animal Infectious Diseases and Zoonoses, Yangzhou, 225009 China

**Keywords:** Goose parvovirus, Muscovy duck parvovirus, Recombination, Inverted terminal repeats

## Abstract

**Background:**

Muscovy duck parvovirus (MDPV) and Goose parvovirus (GPV) are important etiological agents for Muscovy duck parvoviral disease and Derzsy’s disease, respectively; both of which can cause substantial economic losses in waterfowl industry. In contrast to GPV, the complete genomic sequence data of MDPV isolates are still limited and their phylogenetic relationships largely remain unknown. In this study, the entire genome of a pathogenic MDPV strain ZW, which was isolated from a deceased Muscovy duckling in 2006 in China, was cloned, sequenced, and compared with that of other classical MDPV and GPV strains.

**Results:**

The genome of strain ZW comprises of 5071 nucleotides; this genome was shorter than that of the pathogenic MDPV strain YY (5075 nt). All the four deleted nucleotides produced in strain ZW are located at the base-pairing positions in the palindromic stem of inverted terminal repeats (ITR) without influencing the formation of a hairpin structure. Recombination analysis revealed that strain ZW originated from genetic recombination between the classical MDPV and GPV strain. The YY strain of MDPV acts as the major parent, whereas the virulent strains YZ99–6 and B and the vaccine strain SYG61v of GPV act as the minor parents in varying degrees. Two recombination sites were detected in strain ZW, with the small recombination site surrounding the P9 promoter, and the large recombination site situated in the middle of the VP3 gene. The SYG61V strain is a vaccine strain used for preventing goose parvoviral disease. This strain was found to be solely involved in the recombination event detected in the P9 promoter region. Phylogenetic analyses between strain ZW and other classical strains of MDPV and GPV were performed. The results supported the in silico recombination analysis conclusion.

**Conclusions:**

MDPV Strain ZW is a novel recombinant parvovirus, and the bulk of its genome originates from the classical MDPV strain. Two virulent strains and a vaccine strain of GPV were involved in the recombination process in varying degrees.

## Background

Muscovy duck parvovirus (MDPV) and Goose parvovirus (GPV) are etiological agents for Muscovy duck parvoviral disease and Derzsy’s disease, respectively [1]. The Muscovy duck parvoviral disease, also named “three-week” disease, mainly occurs in three-week-old Muscovy ducklings; the disease is characterized by locomotor dysfunction, stunting, abnormal feather development, and death. The mortality rate of the disease ranges from 10% to 80% depending on age [2–5]. Derzsy’s disease is also named “gosling plague” in China and has been primarily described by Fang in 1961; it mainly breaks out in one-month-old goslings with the mortality rate reaching as high as 90% [6–8].

In the up-to-date classification report of International Committee on Taxonomy of Viruses (ICTV), MDPV and GPV are defined as virus variants and have a common species name *Anseriform dependoparvovirus* 1, which is classified into the *Dependoparvovirus* genus in the subfamily *Parvovirinae* [9]. The viral genomes are approximately 5.1-kb single-stranded DNA with equal polarities encapsidated in the viral capsids [10]. Their genomes are flanked by identical inverted terminal repeats (ITR), which consist of 456 nucleotides for MDPV, and 442 nucleotides for GPV [11, 12]. The outside portion of ITR can fold on itself to form a palindromic hairpin structure. The inside portion of ITR, also designated as the D sequence, reversely complements with the D′ sequence in the 3′ ITR, allowing the entire genomic molecule to form a larger range of palindromic structure [13]. ITR contains a terminal resolving site (TRS), Rep protein binding site (RBS), and other transcription factor binding sites, which are required for genome replication, transcription, packaging, and rescue from cloning vector [10, 14, 15].

Their genomes have two open reading frames (ORFs), whereby the genetic organization and nucleotide length are identical for both viruses [10]. The left ORF encodes the viral non-structural protein Rep, whereas the right ORF encodes the structural protein. After alternative splicing of mRNA, the larger Rep1 protein and several smaller Rep proteins are produced [16, 17]. The Rep proteins can bind to the ITR and are involved in genome replication and modulation of downstream P41 promoter [18, 19]. By alternative splicing of mRNA, usage of initiation codon, and protease cleavage, the right ORF produces three structural proteins (VP1, VP2, and VP3), which assemble the viral capsid at a ratio of 1:1:8 [20].

The virulent strain B of GPV and strain FM of MDPV share 82.2% similarity at the genome level [10]. Moreover, the two viruses share 83.0% and 81.5% homologies in Rep1 and VP1 proteins at the nucleotide level, respectively, and 90.6% and 87.6% homologies at the amino acid level, respectively [10]. Irrespective of relatively high homologies, host range difference between both viruses is obvious. MDPV only infects Muscovy ducklings and is not pathogenic to goslings, whereas GPV is pathogenic not only to goslings but also to Muscovy ducklings [1, 4].

Currently, several complete genomic data of GPV strains isolated in various places and years are available, which share high homologies (at least 93%) with each other [21, 22]. However, the complete genomic sequence data of MDPV strains are still very limited and their phylogenetic relationships largely remain unknown. Strain ZW was isolated from a deceased Muscovy duckling, which died from a disease outbreak in 2006 in Zhejiang province, China. In the course of retrospective investigation of the MDPV isolates collected from 1999 to 2006, the genome of ZW was found to possess a different restriction endonuclease digestion pattern from other MDPV isolates. In this study, the entire genome of ZW was sequenced and compared with other classical strains of MDPV and GPV. Strain ZW is a hybrid between the classical MDPV and GPV, with the bulk of its genome originating from classical MDPV, whereas the P9 promoter region and greater part of the VP3 gene from a vaccine strain and two virulent strains of GPV.

## Methods

### Virus propagation and purification

The viral stock of strain ZW was diluted 1:50 with sterile saline containing penicillin (2000 U/ml) and streptomycin (2000 mg/ml) and inoculated into the chorioallantoic cavity of 12-day-old susceptible embryonated Muscovy duck eggs. The procedure for inoculation of fertilized Muscovy duck eggs was approved by the Animal Care and Use Committee of Yangzhou University and performed in accordance with the “Guidelines for Experimental Animals” of the Ministry of Science and Technology (Beijing, China). No specific permits were required for these locations and activities.

The dead embryos were kept at 4 °C for 6 h and the allantoic fluids were pooled and clarified by centrifugation at 11,000×*g* for 20 min. The supernatant was further mixed with chloroform (1/3, *v*/v), shaken vigorously, and then subjected to centrifugation at 11,000×*g* for 20 min. The upper aqueous phase containing the virus was collected and pelleted by ultracentrifugation at 150,000×*g* for 3 h (SW32Ti rotor, Beckman, USA). Finally, the virus-containing pellet was re-suspended in TE buffer (50 mM Tris, 20 mM EDTA, pH 8.0) to 1/50 of the starting volume.

### DNA extraction

The viral DNAs were extracted by the SDS-Proteinase K (Merck, Darmstadt, Germany) incubation method as previously described [23]. The genomic DNA was suspended in STE buffer (10 mM Tris, 1 mM EDTA, 100 mM NaCL, pH 8.0) and annealed by heating to 95 °C for 5 min, followed by slow cooling to 55 °C. The annealed double-stranded DNA of approximately 5.1 kb was examined in 0.8% agarose gel by electrophoresis.

### Genome cloning and sequencing

To facilitate cloning, a pair of linker primers (5′-AGCTTCCATGGG-3′ and 5′-AATTCCCATGGA-3′) was synthesized and annealed, resulting in a linker with adhesive ends of *Hin*dIII and *Eco*RI. By inserting the linker into the plasmid pBluescript II SK (Agilent, Santa Clara, USA), the original *Eco*RV site was replaced by the *Nco*I site, thereby resulting in the plasmid pBSKN [12].

**Fig. 1:**
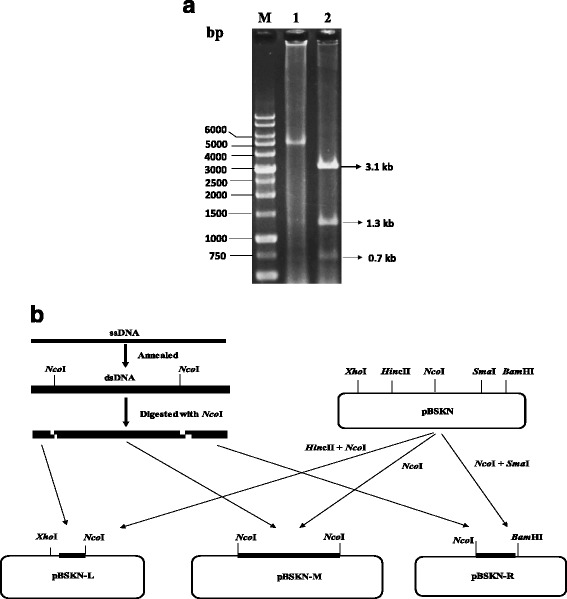
The strategy for cloning the entire genome of strain ZW. **a.** Restriction endonuclease digestion of the extracted genome of strain ZW. 1. The annealed genomic dsDNA of about 5.1 kb; 2. *Nco*I digestion of the genome resulting in 3.1-, 1.3-, and 0.7-kb DNA bands; M: 1-kb DNA ladder. **b.** Schematic diagram of the experimental strategy for cloning three sub-genomic fragments of strain ZW

The extracted genomic DNA was digested with *Nco*I, resulting in a 0.7-kb left fragment, a 3.1-kb middle fragment, and a 1.2-kb right fragment. The digested fragments were separated by electrophoresis and purified with a gel extraction kit (Tiangen, Peiking, China). The 0.7-kb fragment containing the 5′ end ITR was ligated into the *Hin*cII-*Nco*I site of the pBSKN plasmid, thereby generating plasmid pBSKNL (Fig. 1). The middle 3.1-kb fragment was ligated into the *Nco*I site, resulting in plasmid pBSKNM. The 1.2-kb fragment containing the 3′ end ITR was ligated into the *Nco*I-*Sma*I site, generating the plasmid pBSKNR.

The cloned gene fragments were sequenced using an ABI-PRISM3730 automated sequencer and BigDye terminators v3.1 (Applied Biosystems, Foster City, USA). To overcome the difficulties in sequencing the ITRs, the plasmids were double digested with *Sph*I and *Bam*HI or *Kpn*I. The unique *Sph*I site at the loop position of the bubble region allowed us to cut the entire ITR into two halves. The resulting fragments containing half of the ITR were then subcloned into pUC18 for sequencing [24]. The sequences were assembled using SeqManII software included in the Lasergene package 5.0 (DNASTAR, Madison, USA). The complete genome sequence was submitted to GenBank.

### Recombination and phylogenetic analysis

The recombination analysis software SimPlot version 3.5.1 [25] and Recombination Detection Program v.4.43 (RDP4) [26] were both used to detect possible recombinant events that occurred in strain ZW. Simplot analysis was conducted by setting the window width and the step size to 200 bp and 20 bp, respectively. The genomic sequence of strain ZW obtained in this study was used for queries, while putative parental sequences were retrieved from GenBank (Table 1). Different methods implemented in RDP4, including RDP, GENCONV, MaxChi, BootScan, SicCAN, LARD, and 3SEQ were also used to detect recombinant events under default settings for the different detection programs, likely parental isolates, and recombination break points. If a recombination event was supported by at least three methods with a *P*-value <10^−6^ or the recombination score is above 0.6, the recombination event is considered to be true. If the recombination score is between 0.4–0.6, the recombination event is considered to be of fair likelihood. Phylogenetic trees were constructed using the neighbor-joining method in MEGA 6.01 [27].

**Table 1:** Waterfowl parvovirus isolates used in this study for sequence comparison, recombination analysis, and construction of phylogenetic tree

Viral classification	Strain	Pathogenicity	Native host	Geographic origin	Collection date	GenBank accession no.
MDPV	FM	Pathogenic	Muscovy duck	France	n.a	U22967
	P	Pathogenic	Muscovy duck	China	1988	JF926697
	FZ91–30	Vaccine	Muscovy duck	China	1991	KT865605
	YY	Pathogenic	Muscovy duck	China	2000	KX000918
GPV	B	Pathogenic	Goose	Hungary	1967	U25749
	06–0329	Pathogenic	Goose	Taiwan	2006	EU583391
	82–0321	Pathogenic	Goose	Taiwan	1982	EU583390
	VG32–1	Vaccine	Goose	Germany	n.a	EU583392
	SYG61v	Vaccine	Goose	China	1961	KC996729
	LH	Pathogenic	Goose	China	2012	KM272560
	YZ99–6	Pathogenic	Goose	China	1999	KC996730
Recombinant	ZW	Pathogenic	Muscovy duck	China	2006	KY744743

n.a.: not available

### Agar gel precipitation test

To study the antigenic relationship between the recombinant strain ZW and the classical GPV and MDPV strains, agar gel precipitation (AGP) tests were conducted. A mouse monoclonal antibody (mAb) against GPV used in the AGP tests was developed and provided by Professor Guoxiong Dong at College of Veterinary Medicine of Yangzhou University. This mAb can specifically recognize the VP3 protein of GPV in a Western blot test [28]. A 1:20 dilution of the mAb was further diluted by two-fold series in phosphate buffered saline (pH 7.4). The viruses used as antigens in the AGP tests were concentrated by ultracentrifugation as previously described. The gel pattern consisted of a central well 3 mm in diameter containing antigen surrounded by six outer wells of the same diameter containing serially diluted mAb. All the wells were spaced 4 mm apart. Diffusion of antigen and antibody took place at 37 °C and the plates were examined for precipitin lines after approximately 24 h.

## Results

### Genome sequence analysis

After annealing of the single-stranded DNA, a 5.1-kb double-stranded DNA (dsDNA) was observed (Fig. 1a). The dsDNA was digested with *Nco*I, resulting in three fragments of 0.7, 1.3, and 3.1 kb. Subsequently, three sub-genomic fragments were successfully cloned into the pBSKN plasmid for sequencing (Fig. 1b). The obtained sequences were used to assemble the complete genome sequence of strain ZW.

**Fig. 2:**
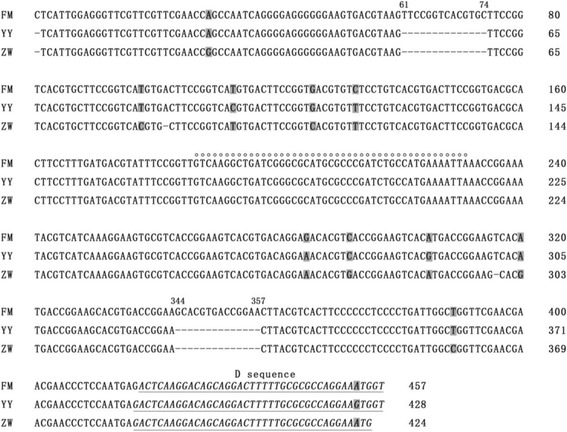
Alignment of inverted terminal repeat (ITR) sequences at the 5′ terminus of the genome of strain FM, YY, and ZW of MDPV. Dashes denote nucleotide deletions. The bubble region of ITR comprises of 45 nucleotides and is indicated by open circles above the letters. The nucleotide differences between three strains are shaded grey. The nucleotides constituting the D region are italicized and underlined, which complement the D′ sequences at the 3′ ITR. The numbers above the alignment denote the nucleotide’s position

The genome of strain ZW consists of 5071 nucleotides—four nucleotides shorter than that of the pathogenic Chinese MDPV strain YY. The ITR consists of 424 nucleotides, among which 387 nucleotides constitute the palindrome and 39 nucleotides constitute the D region (Fig. 2). The D sequence strictly complements the D′ sequence in the 3′ ITR. In comparison to strain FM and FZ91–30 of MDPV, the ITR of strain ZW produces a 14-nucleotide-pair deletions in the stem of the palindrome, which is also observed in the pathogenic strain YY of MDPV [24]. Moreover, strain ZW exhibits additional two nucleotide deletions in the palindromic stem; nevertheless, both deleted nucleotides lie at the base-pairing positions without influencing the formation of a hairpin structure. Sequence comparison of the ITRs with the pathogenic strains of MDPV and GPV showed that strain ZW shared 97.9% nucleotide similarity with the MDPV strain YY but showed lower similarities with the GPV strain LH (85.7%) and YZ99–6 (86.0%), thereby demonstrating that the ITR of strain ZW originates from the classical MDPV.

In the two open reading frames (ORFs), strain ZW shares identical genetic organization with the classical strains of MDPV and GPV. The Rep1 protein of strain ZW consists of 1884 nucleotides, coding for 627 amino acids. Homology comparison showed that the Rep1 protein of ZW displayed a higher nucleotide identity (98.0%–98.7%) with the classical MDPV strains, but a lower similarity (81.8%–83.3%) with the classical GPV strains (Table 2). The VP1 protein of ZW consists of 2199 nucleotides, coding for 732 amino acids. Nucleotide sequence comparison of the VP1 proteins showed that strain ZW displayed almost equal percentages of identity with the classical MDPV strains (89.0%–89.1%) and the classical GPV strains (87.9%–89.4%), whereas percentages of identity with the classical MDPV strains and the classical GPV strains are above 98.5% and 93.5%, respectively (Table 2).

**Table 2:**
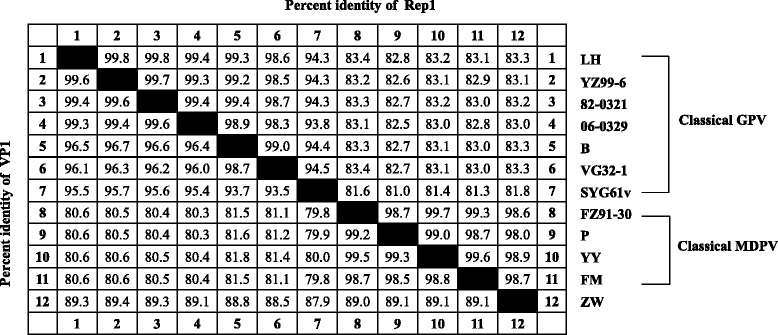
Percentages of identity of nucleotide sequences of the coding proteins Rep1 (upper right) and VP1 (lower left) between strain ZW and other classical MDPV and GPV strains

The genome sequence of strain ZW was deposited in GenBank with the accession number KY744743.

### Recombination analysis

**Fig. 3:**
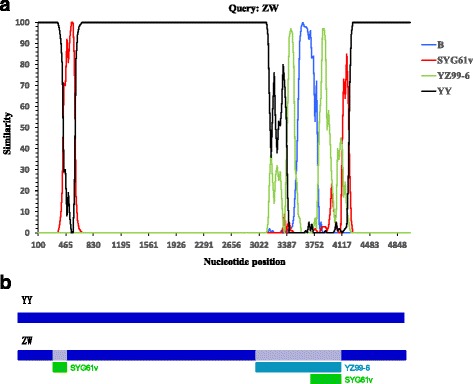
Recombination analysis using Simplot 3.5.1 and RDP 3.4.1. **a.** Similarity plot analysis of full genome sequences of strain ZW, B, SYG61v, YZ99–6, and YY using Simplot. Bootscan analysis was executed with the following parameters: 1000 bootstrap replicates, sliding window of 200 bp, and step size of 20 bp. Strain ZW was used as query. **b.** Recombinant events that occurred in strain ZW were detected by RDP. The first recombination breakpoint began at nucleotide position 424 and ended at nt position 615. The second recombination breakpoint began at nucleotide position 3120 and ended at nt position 4246. In these recombinant events, strain YY is the major parent. SYG61v is the sole minor parent in the first recombinant site

**Fig. 4:**
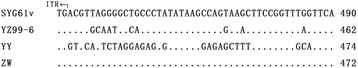
Alignment of partial nucleotide sequences spanning from downstream of ITR to the P9 promoter. Identical nucleotides relative to the SYG61v strain are denoted by dots. The recombinant strain ZW shares 100% nucleotide homology with the vaccine strain SYG61v

Two potential recombination sites that existed in Strain ZW were both detected by the Simplot and RDP software. The first recombination breakpoint in the left side of genome was generated between the YY strain of MPV and the vaccine strain SYG61v of GPV (Fig. 3). Recombination analysis executed with RDP4 showed that the breakpoint started at nucleotide position 456 and ended at position 647, with the highest probability value of RDP method reaching 10^−49^. The major parent was strain YY, and the minor parent was SYG61v. The recombination signal extended from the interior of the 5′ ITR to the 100 nucleotides downstream of the initiation codon in Rep protein. Sequence alignment of 50 nucleotides upstream of this recombination region among YY, YZ99–6, SYG61v, and ZW clearly demonstrated that strain ZW shared 100% identity with the vaccine strain SYG61v, but had variable extents of nucleotide differences with the MDPV strain YY and the GPV strain YZ99–6 (Fig. 4), further confirming the recombination analysis result.

The second recombination breakpoint occurred in the middle of the VP3 gene. Recombination analysis using RDP4 revealed that the second recombination breakpoint started at nucleotide position 3152, which is generally situated at 120 nucleotides downstream of the initiation codon of VP3 protein, and ended at position 4278, covering a successive DNA region of approximately 1100 nucleotides in length (Fig. 3b). The YY strain of MDPV remained the major parent, whereas the two virulent strains (YZ99–6 and B) and the vaccine strain (SYG61v) of GPV acted as the minor parents with the *P*-values ranging from 10^−88^ to 10^−4^. Furthermore, SimPlot analysis also disclosed multiple constitutive recombination signals in the VP3 gene, which were generated between strain YY and the three GPV strains YZ99–6, B, and SYG61v (Fig. 3a). The recombination events were found to mainly occur between strain YY and the virulent GPV strains, whereas only a small portion of recombination region was swapped between YY and the vaccine strain SYG61v. Therefore, locations of the putative recombination sites identified by SimPlot coincided with the recombination analysis result by RDP4.

### Phylogenetic analysis

Based on the recombination analysis results of strain ZW, four genomic fragments were chosen to independently construct phylogenetic trees, among which three fragments are located in the non-recombination regions and one fragment encompasses the identified 1100-bp recombination region of the VP3 gene. Three non-recombination fragments included the carboxyl terminal 1774-bp fragment of the Rep1 gene (Fragment A), and the amino terminal 694-bp fragment (Fragment B) and the carboxyl terminal 405-bp fragment (Fragment C) of the VP1 gene. Moreover, Fragments B and C were adjacent to the 1100-bp recombination fragment (Fragment D).

**Fig. 5:**
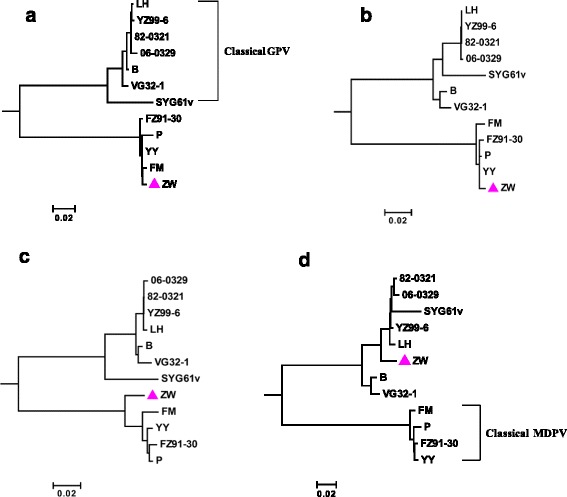
Phylogenetic analysis of strain ZW and other 11 MDPV and GPV isolates. These isolates have complete Rep1 and VP1 gene sequences deposited in GenBank. The neighbor-joining method in MEGA 6.0.6 with 1000 bootstrap replications was used for construction of phylogenetic trees. Phylogenetic trees was constructed based on the C-terminal 1774-bp fragment of the Rep1 gene (**a**), the N terminal 694-bp (**b**) and the C-terminal 405-bp (**c**) fragments of the VP1 gene, and the middle 1100-bp fragment of the VP3 gene (**d**)

In the phylogenetic trees constructed based on the Fragment A, B, and C, strain ZW clustered with all of the classical MDPV isolates and formed a separate branch, distinct from the branch formed by all classical GPV isolates (Figs. 5a, b, and c). In contrast, in the phylogenetic tree constructed based on the Fragment D, strain ZW clustered with the classical GPV members, and all classical MDPV isolates formed another branch (Fig. 5d). Essentially, positional alteration of strain ZW in the phylogenetic trees based on different genomic fragments is in agreement with the in silico recombination analysis result.

### Antigenic analysis by AGP tests

**Fig. 6:**
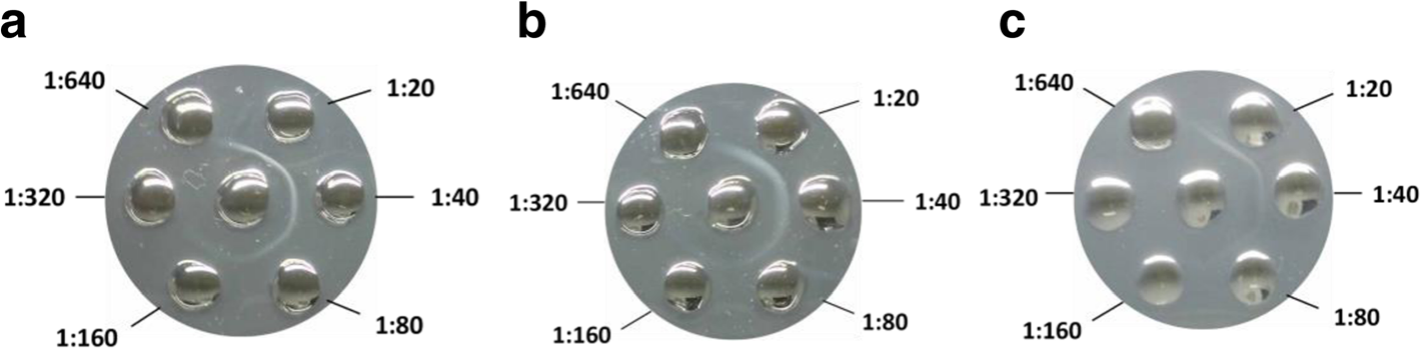
Antigenic comparison between the recombinant strain ZW and the classical GPV and MDPV strains by agar gel precipitation (AGP) tests. A monoclonal antibody (mAb) against GPV was used in the AGP tests. A 1:20 dilution of this mAb was further diluted by two-fold series in phosphate buffered saline (pH 7.4) and placed in the outer wells surrounding the central well containing antigen. The classical GPV strain LH (**a**), the recombinant strain ZW (**b**), and the classical MDPV strain YY (**c**) were used as the AGP antigens, respectively

In the AGP tests, antigens placed in the central wells were the classical GPV strain LH, the classical MDPV strain YY, and the recombinant strain ZW, respectively. A similar mAb titres with 1:160 were obviously observed when strain ZW and LH were used as the AGP antigens (Fig. 6). In contrast, in the AGP test with strain YY as the antigen, the weak precipitin lines were observed and indicated a lower mAb titre with 1:80. The AGP test results demonstrated a closer antigenic relationship between the recombinant strain ZW and the classical GPV strain, which is in agreement with the in silico recombination analysis result.

## Discussion

Attenuated live viruses are involved in genetic recombination with prevailing field strains, thereby resulting in the emergence of novel variants [29, 30]. SYG61v is an attenuated vaccine strain of GPV and combined with the attenuated MDPV strain to prevent potential infection of either GPV or MDPV in Muscovy ducklings. However, usage of the combined vaccine also provides the chance for genetic recombination. In the present recombination analysis, we found that SYG61v not only participated in the recombination event in the P9 promoter region but was also involved in the recombination events that occurred in the VP3 gene. Remarkably, the DNA sequence in strain ZW surrounding the P9 promoter region was wholly replaced with the sequence from strain SYG61v. Considering that strain ZW itself is highly pathogenic to Muscovy ducklings, the biological purpose underlying this recombination event involving a vaccine strain remains to be investigated.

Strain B, the earliest Europe isolate of GPV, was isolated from Hungary in 1967 [8]. In this study, strain B was found to be involved as the minor parent in the recombination events that occurred in the VP3 region of strain ZW. The percentages of identity of the VP1 protein nucleotide sequences between strain B and four other Chinese virulent strains (82–0321, 06–0329, YZ99–6, and LH) ranged from 96.4% to 96.7%, whereas the same analysis among the four Chinese strains showed slightly higher percentages of identity, ranging from 99.3% to 99.6% (Table 2). Involvement of strain B in the recombination events demonstrated that GPV strains from various geographical origins collectively contributed to the generation of the recombinant strain ZW.

The VP3 gene consists of 1605 nucleotides encoding for 534 amino acids, which overlaps with the carboxyl terminus of the VP1 and VP2 proteins. As the major structural protein, the VP3 protein harbors most of the antigenic epitopes distributed on the surface of the viral capsid [10]. In this study, the VP3 gene of strain ZW incorporated approximately 1.1-kb foreign nucleotide sequence from GPV, which ultimately shaped the distinctive antigenicity of strain ZW, different from the classical strains of MDPV and GPV. In addition to the recombination events, several amino acid point mutations were also found to occur in the VP1 protein of strain ZW, all of which are specific for strain ZW and are different from those of the classical MDPV or GPV strains. Point mutations at amino acid positions 450, 485, 493, 548, and 585 were in the recombination region of the VP3 protein, whereas point mutations at positions 79 and 712 were outside of the recombination region. Therefore, the recombination and point mutations contributed to rapid evolution and increased fitness, respectively, and collectively promoted the emergence of the recombinant MDPV strain ZW.

Sequence alignment of ITR indicates the characteristic two-nucleotide deletions that occurred at the palindromic stem in the 5′ and 3′ ITR of strain ZW. ITR not only functions as an origin of genome replication, but also contains several transcription factor binding sites, including E-box, ATF/CREB, and MLTF [10, 24]. These transcription factor binding sites are densely distributed in the 200-bp stem of the palindromic structure of ITR. Although these deleted nucleotides were at the base-pairing positions and did not influence the hairpin structure formation for the remaining nucleotides, the nucleotide deletions shortened the spacing distance between adjacent transcription factors. Whether the nucleotide deletions that occurred in ITR have potential influence on the transcription efficiency and contribute to virulence of strain ZW remain to be further investigated.

Most of viral sequences used for the recombination analysis were obtained through PCR amplification [29–31]. If the PCR templates of the characterized recombinants per se were contaminated with genetic materials from multiple strains, plus degeneration of the designed primers used in PCR amplification, an inauthentic genomic sequence information will probably be generated, which would result in false conclusions in the subsequent recombination analysis. In view of this, we conducted the direct genome cloning strategy by strain ZW by restriction endonuclease digestion. Therefore, the possibility that the genomic sequence of strain ZW has been contaminated with other isolates has been excluded.

For the first time, we presented a detailed description of the genomic characteristics of the novel MDPV strain ZW and confirmed the occurrence of recombination events between GPV and MDPV. Continuous molecular epidemiological investigation should be deployed to assess the incidence of strain ZW-like recombinant viral infection in the field. In view of the widespread usage of live vaccines in the Muscovy duck flocks, direct genome cloning and sequencing against the MDPV isolates is a more reliable method than PCR amplification for acquiring accurate sequence data for recombination analysis. However, whether the traditional bi-combined vaccine currently in use can still provide robust protection for Muscovy ducklings against infection from emerging recombinant MDPV in China remains to be evaluated and clarified.

## Conclusions

Strain ZW is a recombinant MDPV that produces four characteristic nucleotide deletions in the palindromic stem of ITR. Most of the genome of strain ZW, including the ITR sequence, still originates from the classical MDPV strain. Two virulent strains and a vaccine strain of GPV were involved in multiple recombination events detected in strain ZW at different levels. The attenuated vaccine strain SYG61v acted as the only minor parent, thereby contributing to the recombination event that occurred in the P9 promoter region. Considering that strain ZW emerged in 2006, we deduced that the recombinant MDPV has been co-existing with the classical MDPV strains for a long time in the field and has constantly threatened Chinese Muscovy duck industry.

## Abbreviations

GPV: Goose parvovirus
ITR: Inverted terminal repeats
Kb: kilobase
MDPV: Muscovy duck parvovirus
Nt: nucleotides
ORF: Open reading frame
